# Timing-dependent LTP and LTD in mouse primary visual cortex following different visual deprivation models

**DOI:** 10.1371/journal.pone.0176603

**Published:** 2017-05-17

**Authors:** Yatu Guo, Wei Zhang, Xia Chen, Junhong Fu, Wenbo Cheng, Desheng Song, Xiaolei Qu, Zhuo Yang, Kanxing Zhao

**Affiliations:** 1 Tianjin Eye Hospital, Tianjin Eye Institute, Tianjin Key Lab of Ophthalmology and Visual Science, Tianjin, China; 2 Department of Ophthalmology, The TEDA International Hospital, Tianjin, China; 3 Department of Ophthalmology, the Second People’s Hospital of Jinan, Shandong, China; 4 Key Laboratory of Bioactive Materials, Ministry of Education, College of Life Science, Nankai University, Tianjin, China; 5 College of Medicine, Nankai University, Tianjin, China; Universidade do Estado do Rio de Janeiro, BRAZIL

## Abstract

Visual deprivation during the critical period induces long-lasting changes in cortical circuitry by adaptively modifying neuro-transmission and synaptic connectivity at synapses. Spike timing-dependent plasticity (STDP) is considered a strong candidate for experience-dependent changes. However, the visual deprivation forms that affect timing-dependent long-term potentiation(LTP) and long-term depression(LTD) remain unclear. Here, we demonstrated the temporal window changes of tLTP and tLTD, elicited by coincidental pre- and post-synaptic firing, following different modes of 6-day visual deprivation. Markedly broader temporal windows were found in robust tLTP and tLTD in the V1M of the deprived visual cortex in mice after 6-day MD and DE. The underlying mechanism for the changes seen with visual deprivation in juvenile mice using 6 days of dark exposure or monocular lid suture involves an increased fraction of NR2b-containing NMDAR and the consequent prolongation of NMDAR-mediated response duration. Moreover, a decrease in NR2A protein expression at the synapse is attributable to the reduction of the NR2A/2B ratio in the deprived cortex.

## Introduction

It is known that visual experience modifies cortical circuits in the primary visual cortex through synaptic plasticity during a critical period[1,2]. Visual cortical plasticity has conventionally been ascribed to Hebbian or correlation-based mechanisms such as NMDAR-dependent LTP and LTD[3–8]. Specifically, spike timing-dependent plasticity (STDP)[9–12], in which the temporal order of pre- and postsynaptic neuronal activity is critical for the direction of change in synaptic weights, has emerged as a potential mechanism for experience-dependent changes in the neural circuit, including map plasticity of the visual cortex[13–15]. For STDP models at excitatory synapses, the induction of timing-dependent LTP (tLTP) in V1 requires the glutamate binding of NMDA receptors (NMDARs) with the concomitant arrival of a back-propagating action potential (BAP) into the postsynaptic dendrite[16,17]. Additionally, postsynaptic NMDAR activation is required for the induction of tLTD in rodents (ages ≥ P21)[18–20]. Thus, NMDA receptors are considered coincidental detectors of tLTP and tLTD elicitation[20].

Sensory deprivation during a critical period perturbs both the structure[21–23] and function[24–28] of the primary visual cortex (V1), such as dark exposure[29] and monocular deprivation[30–32]. These two visual deprivation paradigms alter various elements of incoming sensory information in the visual cortex. In dark exposure, the complete lack of visually driven activity prevents normal maturation of all functional properties of visual cortical neurons[33,34]. On the contrary, monocular deprivation, using a monocular lid suture, allows diffuse light penetration through the eyelids, thus producing some degree of cortical activation[35,36]. In contrast to the decelerating visual development in dark-exposed mice, monocular deprivation during early postnatal life causes decreases in visual acuity and synaptic depression in the deprived eye[37,38]. Although their effects on cortical neurons vary, these two types of visual deprivation reduce the ratio of GluN2A:GluN2B-containing NMDARs in the deprived cortex[39]. Our hypothesis proposes that the switch in NMDA receptor subunits from the deprived visual cortex in MD mice reshapes the temporal window for STDP induction. This theory compels us to investigate how different forms of sensory deprivation modify the induction of tLTP and tLTD at L4 to L2/3 pyramids in monocular V1 (V1M).

Here, we found that 6-day visual deprivation with either dark exposure or monocular deprivation extended the temporal window for both timing-based LTP and LTD similarly, which is different from the broader LTD window that is at least three and half times longer than the timing window for LTP in the deprived somatosensory cortex.

## Materials and methods

### Animals

Wild-type C57BL/6 mice were maintained on a normal light cycle (12 h: 12 h light/dark) until they were 3 weeks old. Food and water were provided *ad libitum*. Mice of either sex were used in this study. All procedures were approved by the Institutional Animal Care and Use Committee of Nankai University.

### Monocular deprivation and dark exposure

Monocular deprivation was performed by suturing the right eyelid of mice between P21-P24 under 2–3% isoflurane anesthesia. The eyelid margins were trimmed and sutured using a 6–0 vicryl stitch. Lomefloxacin ointment was applied to prevent infection. After recovery from anesthesia, MD mice were housed in a normal light cycle for 6 days. Following surgery, the mice were monitored daily to ensure the lids remained closed and uninfected. Animals whose eyelids reopened and mice with corneal opacities or signs of infection were excluded from further experiments. For the dark-exposure experiments, mice were moved into a dark room for 6 days and then sacrificed under infrared illumination with isoflurane anesthesia.

### Cortical slice preparation

Visual cortical slices were prepared as described previously[24,40]. Briefly, mice were sacrificed by decapitation following an overdose of isoflurane. Brains were transferred into an ice-cold dissection buffer containing the following (in mM): 212.7sucrose, 5 KCl, 1.25 NaH2PO4, 10 MgCl2, 0.5 CaCl2, 26 NaHCO3, 10 dextrose, bubbled with 95% O2/5% CO2 (pH 7.4). Then, 300 μm coronal slices were cut using a vibrating microtome (VT1000S; Leica). Slices were removed to normal artificial cerebrospinal fluid (ACSF) for at least half an hour prior to recording (composition: mM: 124 NaCl, 5 KCl, 1.25 NaH2PO4, 26 NaHCO3, 10 dextrose, 1 MgCl_2_, and 2 CaCl_2_, saturated with a mixture of 5% CO2/95% O_2_).

### Electrophysiology

**Fig 1:**
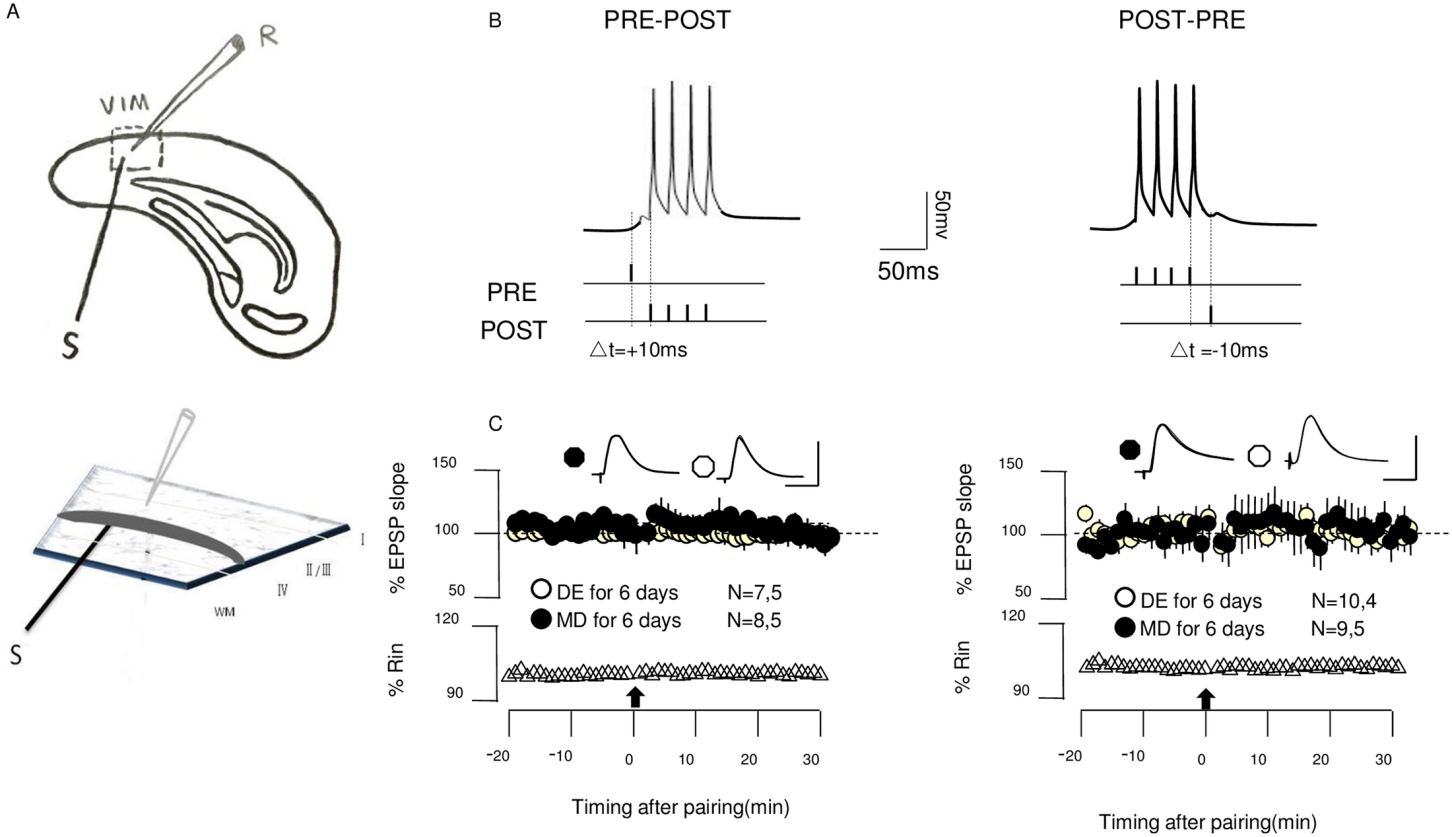
Timing dependent LTP and LTD induction. **(A)** Experimental schematics. EPSPs were recorded in layer II/III pyramidal cells of V1M by stimulating the underlying layer IV. **(B)** Conditioning paradigm. During each pairing epoch (200 at 1 Hz), stimulation of one pathways was paired with a postsynaptic burst of four action potentials (100 Hz). **(C)**In control ACSF, the pairing paradigm (arrow) does not induce tLTP (left) and tLTD(right) in cells from dark-exposed (open circles) or monocular deprived mice (filled circles). Representative averaged responses of EPSPs are recorded before (thin line) and 30min after conditioning (thick line) (superimposed traces). Membrane input resistance (Rin) is displayed below. Plotted data is average± SEM. Calibration:10mV,5msSuperimposed traces are averages of 10 consecutive responses recorded right before(thin line) and 30 min after conditioning (thick line).

Visualized patch-clamp recordings were obtained from layer 2/3 regular-spiking pyramidal neurons in the V1M from the deprived hemispheres as previously reported (Fig 1A) [41]. In brief, V1Ms were identified using a mouse brain atlas. Slices were taken from 1.0mm anterior to lambda and 2.5mm lateral from the midline. The shape and morphology of the white matter (WM) was used as an additional landmark. Care was taken to record the neurons located in the expected center of the V1M to avoid mistaken neurons on the boundaries. Neurons were visualized using infrared-differential interference contrast optics. Layer 2/3 pyramidal cells were identified based on laminar position, morphology, synaptic properties and firing properties. Morphology and location of neurons were further confirmed by biocytin fills *post hoc*. Whole-cell current clamp recordings were obtained using patch pipettes (4–6 MΩ) filled with intracellular solution containing (in mM) 130(K) Gluconate, 10 KCl, 0.2 EGTA, 10 HEPES, 4 (Mg)ATP, 0.5 (Na)GTP, and 10 (Na) Phosphocreatine (pH adjusted to 7.25 with KOH, 280–290 mOsm). Recordings were generated using a Multiclamp 700A amplifier (Molecular Devices). Only cells with membrane potentials more negative than −65 mV, series resistance < 20 MΩ, and input resistance greater than 100 MΩ were included for analysis. Cells were discarded if the input resistance changed >20% during the recording, with the exception of changes during bath application of the agonists. The resulting signals were filtered at 2 kHz and captured at 10 kHz using the pCLAMP10.0 software (Molecular Devices Inc., Sunnyvale, CA)

EPSPs were elicited with a two-contact luster electrode (125 μm diameter; FHC, Bowdoin, ME). The stimulus intensity to L4 was adjusted to evoke 4–6 mv responses with 0.2 ms paired pulses (100 ms interval) delivered every 20 s. The slope of the EPSP (the first 2 ms) was calculated as the mean baseline slope from 30 consecutive sweeps. After 10 minutes of drug application, presynaptic EPSPs evoked by layer 4 stimulation were paired with postsynaptic burst firing evoked by four brief suprathreshold current pulses (2 ms duration, 10 ms apart, 1.5 times threshold current) (Fig 1B). Associative pairing consisted of 200 pairing periods delivered at 1 Hz. Following the condition stimuli, the EPSPs evoked by resumed test stimuli were recorded for another 30 mins. One cell per slice was used.

Identification of NMDA- receptor-mediated EPSCs was based on their voltage dependency and their responses to glutamate antagonists. The NMDAR-mediated EPSCs (NMDA-EPSCs) clamped at+40 mV were pharmacologically isolated in ACSF containing 4 mMCaCl_2_, 4 mM MgCl_2_, 20 μM CNQX (Sigma) and 2.5 μM Gabazine. To evaluate the deactivation kinetics of the NMDAR-EPSCs, 30–50 evoked traces were averaged and normalized. The current decay of NMDA-EPSC was fit using a double-exponential formula:

$$I\left(t\right)=I_{f}\text{exp}\left(-t/T_{f}\right)+I_{s}\text{exp}\left(-t/T_{s}\right)$$

where If and Is are the amplitudes of the fast and slow decay components and τf and τs are their respective decay time constants. Then, we calculated the weighted decay time constant:

$$\tau_{\omega }=\tau_{f}\left(I_{f}/\left(I_{f}+I_{s}\right)\right)+\tau_{s}\left(I_{s}/\left(I_{f}+I_{s}\right)\right)$$

### Synaptoneurosome preparation

C57/BL6 mice (P21-P24) underwent euthanasia. The deprived primary visual and frontal cortices were rapidly dissected in cold dissection buffer (as mentioned above) and immediately put in an ice-cold synaptoneurosome buffer (10 mM Hepes, 2.0 mM EDTA, 2.0 mM EGTA, 0.5 mM DTT, 0.1 mM PMSF, 10 mg/liter leupeptin, 50 mg/liter soybean trypsin inhibitor, 100 nM microcystin). Then, primary visual cortical regions containing the V1M were microdissected. Tissue was homogenized in a glass-glass tissue homogenizer (Samro, Shanghai). A subcellular fractionation procedure (synaptoneurosomes) was implemented to procure synaptic protein samples[39,42,43]. The procedure was executed as follows: the homogenate was passed through a 5-μm-pore hydrophobic mesh filter and centrifuged for 10 min at ×1000 g to obtain the synaptic fraction of the membrane. The resulting pellets were resuspended in boiling 1% SDS and stored at -80°C.

### Immunoblotting

Western blotting was used to quantify protein expression. Equal amounts of synaptoneurosome protein (20 μg), using the bicinchoninic acid (BCA) assay (Pierce, Rockford, IL,USA), were separated on 7.5% SDS-PAGE gels (Bio-Rad) and transferred to PVDF membranes (Millipore,Billerica, MA, USA). After blocking in 5% nonfat dry milk (wt/vol) for 1 h, the membrane was incubated with subunit-specific anti-NR2A (rabbit polyclonal l; 1:1000, ThermoFisher, PA5-35377) and anti-NR2B (rabbit polyclonal; 1:1000, ThermoFisher, 71–8600) in TBS-T overnight at 4°C. After 3 washes with TBS-T, the membranes were incubated in respective HRP-conjugated secondary antibodies for 1 h (1:3500, Sigma). To monitor the loading and blotting of an equal amount of protein, the membranes were incubated with an anti-actin antibody (1:500, Sigma-Aldrich). Immunoreactive bands were visualized by enhanced chemiluminescence (ECL) (Amersham, Pharmacia Biotech, Piscataway, NJ, USA) and exposed to autoradiographic film (X-Omat, Kodak, Rochester, NY, USA). The ECL-exposed films were quantified by comparing the intensity of the bands on a western blot using Image-Pro Plus 6.0 (Media Cybernetics Inc. Silver Spring, MD, USA).

Data are expressed as a ratio of optical densities from sequential probes of the same immunoblot, thus circumventing the need to normalize the results to that of β-actin. The blots from DE or MD animals were normalized to the expression of the age-matched, normally reared protein samples.

### Drugs

D-(-)-2-AMINO-5-PHOSPHONOPENTANOICACID (D-AP5) and Ifenprodil (NR2B specific antagonist) were obtained from Tocris Bioscience,(Avonmouth, Bristol, UK). Isoproterenol, Gabazine, and 6-Cyano-7-nitroquinoxaline-2,3-dione (CNQX) were purchased from Sigma Aldrich(St.Louis, MO,USA).

### Statistical analysis

All values are presented as the average ± SEM. The magnitude of plasticity was measured as the average of the last 10 min of recording (initial slope of the EPSP), beginning 20 min after conditioning stimulation. The significance of LTP and LTD was assessed using the paired Student’s t-test. Other comparisons were completed using Student’s t-test or the ANOVA test.

## Results

In this study, our goal was to compare the effects of 6-day deprivation on the induction of STDP in the primary visual cortices of mice following monocular deprivation and dark exposure. Unlike dark exposure, monocular deprivation in critical periods disrupts the visual experience and causes a loss of visual responsiveness throughout the primary visual cortex and shifts in ocular dominance[6,38,44]. One important difference identified was the absence or presence of competitive interactions[41]. To avoid binocular competition and simplify the experimental condition, we focused on the induction of STDP in the monocular region of V1.

### 1. Timing-dependent LTP and LTD is regulated by neuromodulation in the deprived visual cortex

As permissive gatekeepers, neuromodulators were needed for STDP induction in L4-L2/3 synapses in V1[19,45], including pyramid cells from brief DE mice[24]. To determine the neuromodulators’ effects on STDP induction in cells from MD mice, we examined the EPSP before or after a burst of postsynaptic action potentials for 10 ms without neuromodulator application.

Consistent with previous studies performed in the absence of neuromodulators[19,24,45], we failed to induce tLTP in the V1M of 6-day MD and DE mice. (MD: 98.2% ± 4.6% of baseline at 30 min after the pre-then-post conditioning, n = 8 slices, 5 mice, paired t-test p = 0.711; DE: 98.4% ± 3.4%, n = 7 slices, 5 mice, paired t-test p = 0.34, respectively; Fig 1C). Neither of the pairings induced significant tLTD (MD 101.09% ± 10% of baseline at 30 min after the post-then-pre conditioning, n = 9,5, p = 0.92; DE 98.9% ± 5.9% of baseline at 30 min, n = 10,4, p = 0.87; Fig 1C). Thus, under these experimental conditions, the inputs to the pyramidal cells were not modified by standard STDP protocols. β-adrenergic receptors (coupled to Gs) and α1-adrenergic receptors (coupled to Gq/11) combined with the STDP paradigm were required to enable the induction of tLTP and tLTD in cells from MD mice.

### 2. tLTP and tLTD induced in cells in the deprived primary visual cortex

Partial sensory deprivation dramatically changes the sensory map in the rodent primary somatosensory cortex (S1) and visual cortex[14]. In S1, a deprived sensory input, such as a trimmed whisker, extended the tLTD window three times more than the narrowed tLTP window. However, whether MD influences the temporal precision of tLTP and tLTD in V1M has not been explored.

**Fig 2:**
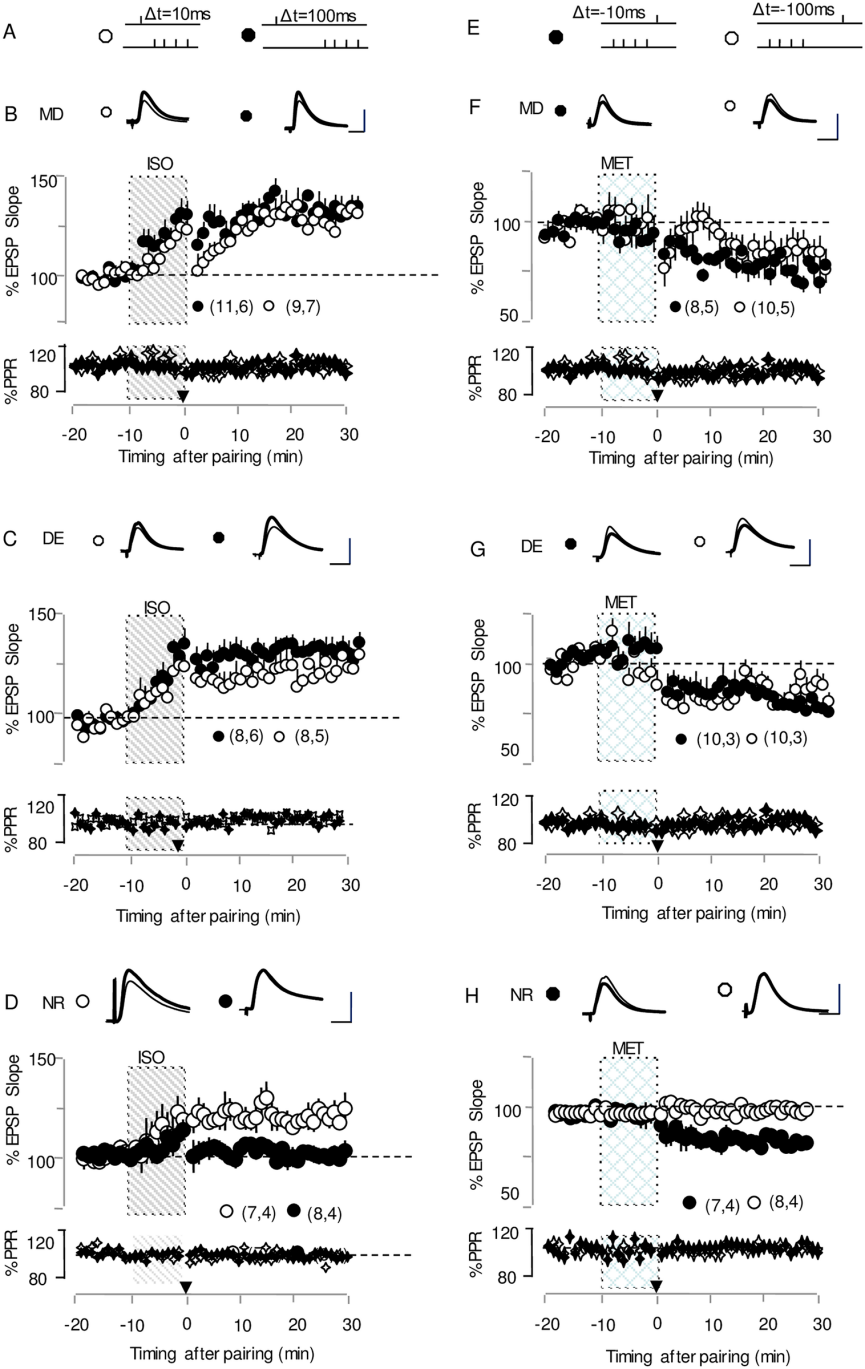
Both monocular deprivation and dark exposure expand the temporal windows for tLTP and tLTD. (A) Conditioning paradigms for tLTP; (E) Conditioning paradigms for tLTD; (B) Bath application of the β-adrenergic agonist isoproterenol (Iso:10 μM, gray bar) increases EPSP slope (open circles) and allows induction of LTP with pairing at +10 ms (filled circles) and +100ms(open circles) in cells from monocular deprived mice. (C)Pairing at +10 ms (open circles) and +100ms (filled circles) in the cells from dark-reared mice result in the induction of LTP. (D) In cells from normal reared mice, isoproterenol promotes the induction of tLTP when pre-then-post delay is 10 ms, but not when it is 100ms. (F) In the presence of the α1 adrenergic agonist methoxamine (Metox: 5 μM for 10 min, grid bar) pairing with -10 ms (filled circles) and -100ms (open circles) induces LTD in cells from monocular-deprived mice. (G)Bath application of the α1 adrenergic agonist methoxamine allows induction of LTD with -10ms (solid circles) or −100 ms (open circles) pairing in cells from dark-reared mice. (H)In cells from normal reared mice,tLTD can be induced with methoxamine when post-then-pre delay is -10ms,but no tlTD is observed when the delay is -100ms. Traces (top) are average of ten consecutive responses recorded before (thin) and after pairing (thick). Note no Changes in pair pulse ratio (PP Ratio) (Bottom graph) for pairing. Plotted data is average ± SEM; Calibration:6 mV, 10 ms.

To address this question, we monitored the pre-then-post spike timing sequences with varied intervals to evaluate the impact of 6-day MD on the temporal window for t-LTP. As shown in Fig 2B, in the presence of Iso, we observed that the pre-then-post stimulus pairing at the L4-L2/3 synapses produced t-LTP in cells from the deprived visual cortices of monocular-deprived (MD) mice with delays of 10 ms and 100 ms. (MD +10 ms: 129.7±4%, n = 11,6, p<0.001; +100 ms:121.8±4.0%, n = 9,7, p<0.001; Fig 2B, Figure B in S1 Fig). Together, these data indicated that monocular deprivation broadened the timing window for tLTP, which was unexpectedly different from the shortened tLTP temporal windows in deprived S1.

We further assessed the tLTP induction in the primary visual cortex slices from 6-day DE mice under the same conditions (pre-before-post pairing). Significant tLTP occurred following pairing paradigms in both delays (DE + 10 ms: 123.5±5.0%, n = 8,6, p<0.01; Δt = + 100 ms:122.7± 5.1%, n = 8,5, p<0.01; Fig 2C, Figure A in S1 Fig). Consistent with our earlier report, in the cortical slices from 6-day DE mice, robust t-LTP could be produced with longer positive timing delays similar to 2-day DE mice.

To investigate whether brief monocular deprivation affects the timing window for tLTD, our tests were conducted in the V1M from the contralateral visual cortex of the deprived eye. Under the post-before-pre paradigm, stimulus pairing at the L4–L2/3 synapses produced long-term depression when the delays were -10 ms and -100 ms (-10 ms: 78.9±4.0%, n = 8,5, p < 0.001; -100 ms: 86.5±6.4%, n = 10,5; Fig 2F, Figure B in S1 Fig), respectively. Similar to MD mice, our tLTD protocol successfully prompted plasticity in DE mice with delays of -10 ms and -100 ms (-10 ms: 77.5±3.6%, n = 10,3, p < 0.001; -100 ms: 84.2±5.0%,n = 10,3; Fig 2G, Figure A in S1 Fig).

For comparative purposes, we observed that the pre-then-post stimulus pairing at L4-L2/3 synapses produces tLTP in cells from normally reared (NR) mice with a delay of 10 ms. In contrast, LTP was not induced by paired stimulation with a 100 ms delay (+10 ms: 123.4±4.2%, n = 7,4; +100 ms: 101.1±3.9%, n = 8,4; p = 0.002 Fig 2D). tLTD was induced under the post-then-pre spiking timing sequences with a 10 ms delay, but this was not observed when the delay was 100 ms (-10 ms: 77.4±4%, n = 7,4; p<0.001; -100 ms: 98.6±3%, n = 8,4; p = 0.37 Fig 2H). Collectively, these data demonstrate that similar to DE mice, the integration windows for both tLTP and tLTD were extended in the L4-L2/3 pathway in the V1M from MD mice.

### 3. Expression and functional properties of NMDAR subunit composition modified by deprivation

**Fig 3:**
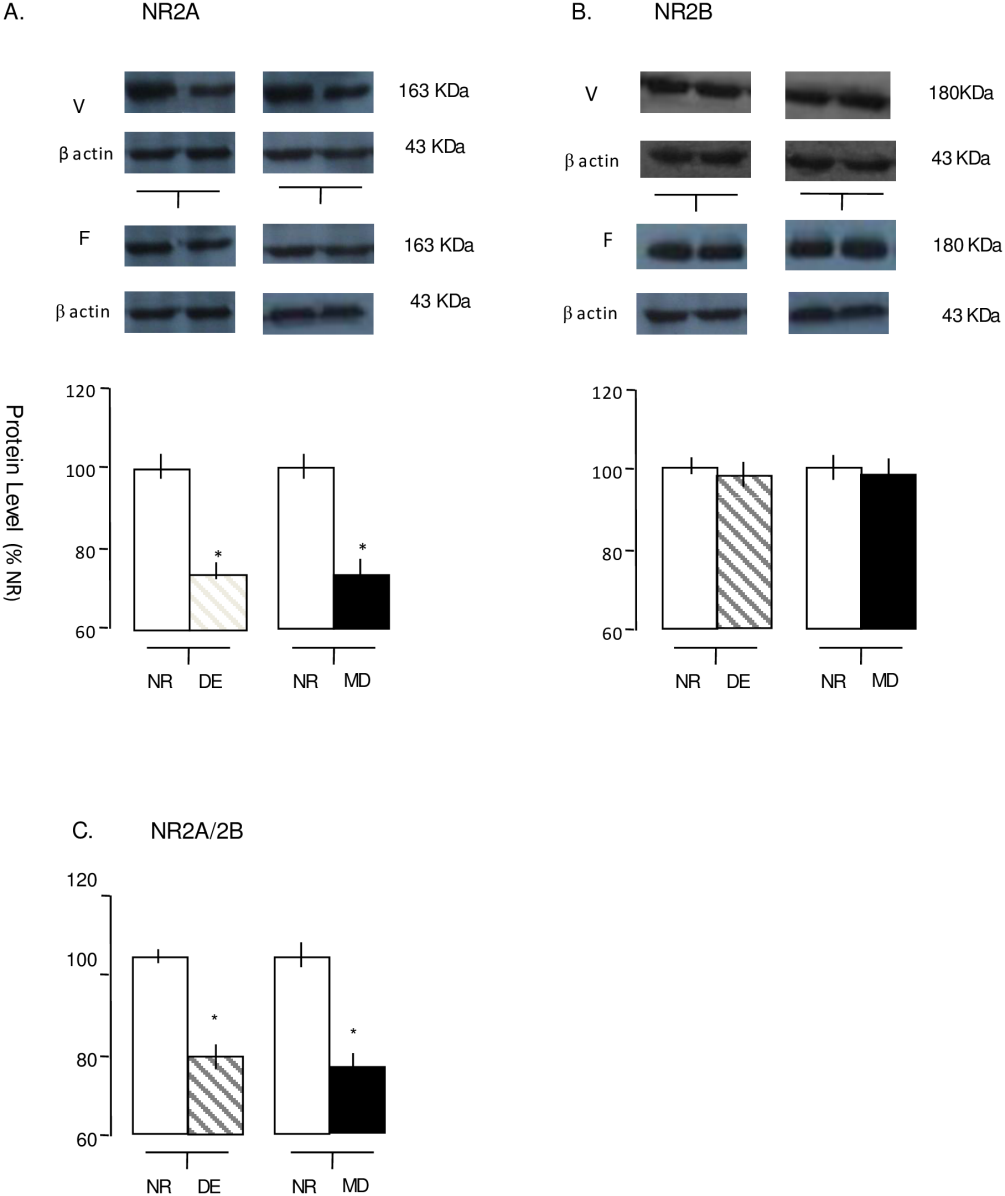
DE and MD modify NMDAR subunit composition in synaptoneurosomes from mice visual cortex. (A-B) top: Representative immunoblots of synaptoneurosome samples prepared from frontal (F) and visual (V) cortices of mice that were normal-reared (NR), dark-exposured (DE) and monocular deprived mice. Blots were probed with subunit-specific antibodies for NR2A (Fig A), NR2B(Fig B). Bottom: Summary of immunoblot analysis of synaptoneurosome samples prepared from Visual cortices (V) of NR and DE mice or NR and MD mice. NR2A protein levels in visual cortex weresignificantly reduced after 6 days of DE (*t*-test p<0.01)or MD(*t*-test p<0.001)(Fig A). NR2B protein were not modified by 6 days of DE *(t*-test p>0.1)or MD *(t*-test p>0.6)(Fig B). Data are expressed as mean values ± SEM normalized to the average NR protein level, n = 8 from 4 animals for each group. (C) The NR2A/2B ratio is reduced in the deprived visual cortex from mice that were under dark exposure *(t*-test p<0.05)or and monocular deprivation *(t*-test p<0.05).

To investigate the effects of different sensory deprivation on the visual cortical NMDAR subunit composition, synaptoneurosomes were prepared from the V1M of mice after dark exposure or monocular deprivation and their light-reared age-matched controls. These questions were addressed by quantifying changes in NMDA receptor subunits (NR2A and NR2B) in the deprived visual cortex. Because NMDAR subunit expression in the frontal cortex was only slightly influenced by visual experience, the frontal cortex served as a within-animal control tissue for animal-to-animal variability. The data presented here are expressed as relative intensities of the NR value. First, we found that the levels of NR2A and NR2B expression in the contralateral visual cortices of MD juvenile mice were significantly different from the normal values. A significant decrease in NR2A (Fig 3A; 73.2± 3.3% of NR, t-test, p < 0.001) rather than an increase in NR2B (Fig 3B; 97.6± 3.8% of NR, t-test, p = 0.66) was observed in the visual cortex contralateral to the deprived eye. An index of NR2A:NR2B expression was calculated for normally reared and monocular-deprived mice, which was significantly reduced at 6 days following MD (Fig 3C, 79.1± 6.03% of NR, t-test, p = 0.002). To confirm that the effect of MD on NMDAR composition was specific to MD, we quantified the changes in the expression of the NMDA receptor subunits in both sides of visual cortices from normal controls. The levels of NR2A and NR2B expression were similar between the left and right sides. Correspondingly, no changes were found in the NR2A/2B ratio (data not shown, p = 0.75, p = 0.82, and p = 0.58 for NR2A, NR2B, and NR2A/2B ratio, respectively).

These changes in NR2A and NR2B expression are remarkably similar to the changes seen in 6-day dark exposure mice. In the visual cortices from 6-day DE animals, there was significantly less NR2A (Fig 3A 73.7± 3.3% of NR t-test, p <0.01) protein expression in synaptoneurosomes than in NR controls according to quantitative immunoblotting. The level of NR2B was slightly reduced with no statistically significant difference (Fig 3B 98.4± 3.9% of NR t-test, p = 0.38). Analysis of the visual cortex data indicates that the decrease in the NR2A/2B ratio is significant at 6 days of dark exposure.(Fig 3C, 76.1+ 3.9% of NR, t-test, p < 0.001).

In general, these data indicated that the expression of NMDAR subunit composition induced specific changes following sensory deprivation. A 6-day deprivation-induced reduction of the NR2A/2B ratio can be achieved by decreasing NR2A expression. The alterations detected in the synaptoneurosome provided a structural basis in preparation for changes in functional synaptic NMDARs.

**Fig 4:**
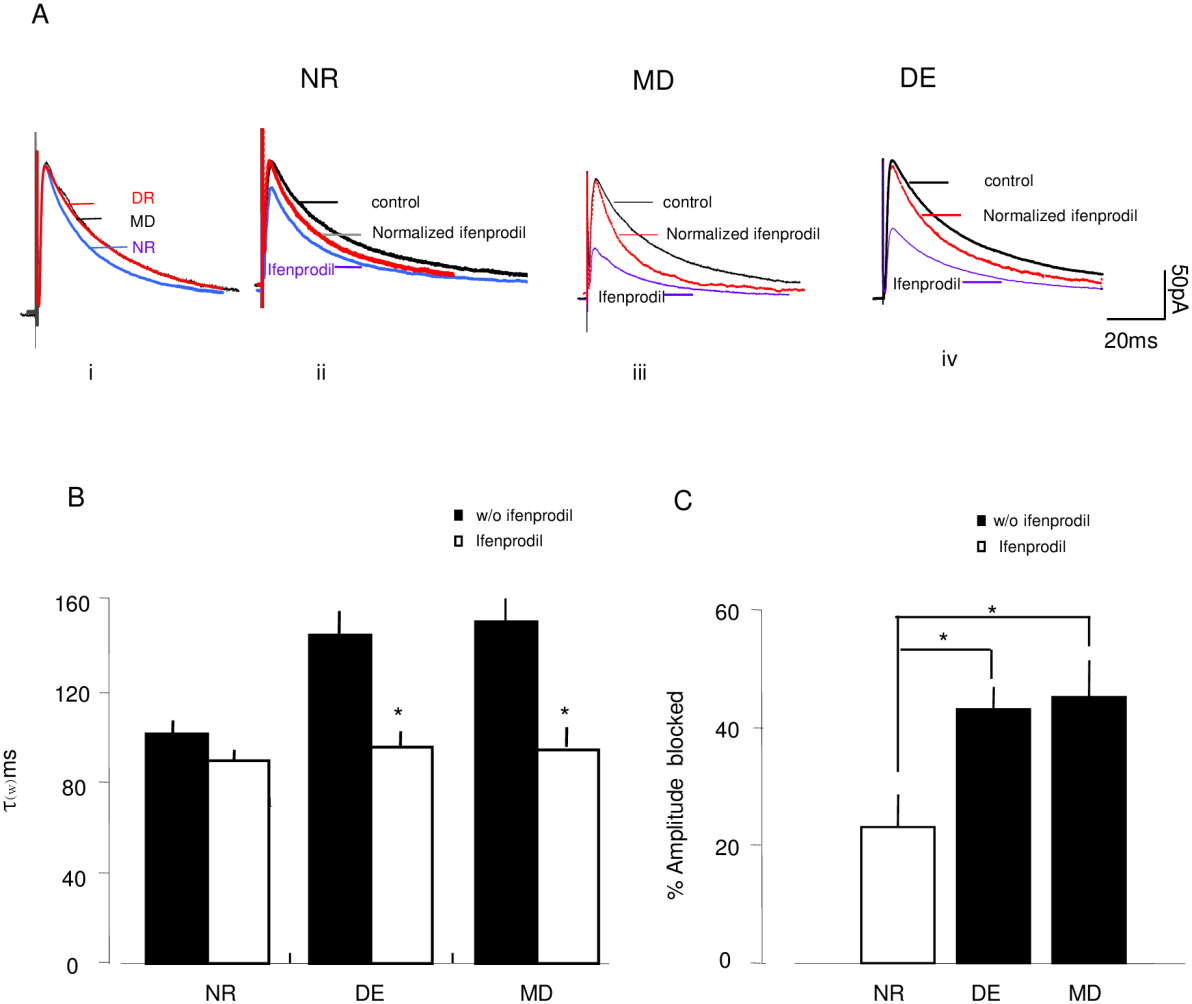
Visual deprivation increases the fraction of NR2b containing NMDAR-mediated response. (A) Overlay of averaged and normalized NMDAR-EPSCs in visual cortex with or without NR2B antagonist ifenprodil application (Ifenprodil: 3 μM) i. Control response from NR,DE and MD mice. The traces are normalized to the peak amplitude in control ASCF and are the average of the responses that all cell recorded in each rearing condition; Normalized NMDAR-EPSCs recorded before and after ifenprodil application in cells from NR mice (ii), MD mice (iii), DE mice (iv). (B) Average Changes in NMDAR-EPSC durations after ifenprodil application. (C) Average changes of the amplitude of the NMDAR-EPSCs blocked by ifenprodil.

Another well-documented consequence of visual experience deprivation is a longer duration of NMDAR response due to a decreased NR2A/NR2B fraction[43]. To confirm the effect of 6-day MD on functionality alterations in the NMDAR subunit, we examined the amplitudes and decay kinetics of NMDAR-mediated currents and ifenprodil sensitivity in layer 2/3 pyramidal cells from the V1M (Fig 4A). Normalization of the NMDAR-mediated EPSCs recorded in cells from the contralateral visual cortices of 6-day MD mice (n = 8,5) and NR mice occurred with the application of NR2B-selective antagonists (3 mM ifenprodil). Compared to NR controls, 6-day MD caused a significant increase in the duration of NMDAR-EPSCs and increased their susceptibility to ifenprodil, which was quantified as a weighed time constant (τw, see Methods) (τw in ms. MDcontrol: 149.10±7.34; MDifenprodil: 93.59±3.16; n = 8,5; NR control: 99.90±6.8; NRifenprodil: 89.3±4.0, n = 8,5; 2-way ANOVA: p = 0.017 for rearing condition; p = 0.009 for interaction with the drug; Fig 4A and 4B). Additionally, the increased amplitude of NMDAR-EPSC after 6-day MD was blocked by ifenprodil more than in normal controls (MD: 45.3±6.2%, n = 8,5; NR: 23.0±5.5, n = 8,5, t-test: p = 0.009 Fig 4A–4C). Based on our studies of EPSC kinetics and ifenprodil sensitivity, these experiments revealed that the synaptic consequences of monocular deprivation are increased and prolonged NMDAR-EPSCs largely mediated by the NR2b-containing NMDAR.

Additionally, we noticed that 6-day dark exposure changed NMDA receptor function by enhancing the fraction of NR2b functionality at the synapse. The amplitude of the NMDAR-EPSCs, blocked by ifenprodil, was increased after 6-day DE (DE: 43.2±4.5%, n = 8,4; NR: 23.0±5.5, n = 8,5. t-test: p = 0.004; Fig 4A–4C). Simultaneously, 6-day DE prolonged the NMDAR-EPSC durations. Moreover, the durations were significant shortened by ifenprodil (τw in ms. DEcontrol: 143.88±10; DEifenprodil: 94.44±7.15 n = 8,5; 2-way ANOVA: p = 0.020 for rearing condition; p = 0.001 for the interaction with the drug; Fig 4A and 4B). It was apparent that robust NMDAR functional changes at visual cortical synapses, including prolonged duration of NMDAR currents, occur following visual deprivation.

### 4. A shift in the NR2A/NR2B ratio influences the integration window of the deprived visual cortex

It is widely believed that t-LTP induction relies on the interaction of BAPs with calcium influx through postsynaptic NMDARs[16,46]. As an important coincidental detector of tLTP, the switch in the NMDAR subunit may influence the properties of tLTP induction. Considering the aforementioned delayed shift in NMDAR subunit expression and the changes of its functional properties, the extended temporal window for tLTP was closely related to the changes in NMDAR decay kinetics based on an increased NR2B-containing NMDAR fraction following sensory deprivation[47,48]. To verify this speculation, we conducted the following experiments.

Before exploring this possibility, we first determined the loci of tLTP and tLTD expression in MD or DE mice. The paired-pulse ratio (PPR) was typically correlated with the initial release probability[49] and changed with presynaptically expressed forms of synaptic plasticity at cortical synapses. We examined the paired-pulse ratio before and after tLTP and tLTD stimuli protocol. The pairing paradigm did not affect the PPR (p >0.15, in all cases), which indicated that both LTP and LTD were unlikely to be mediated by changes in release probability (Fig 2)

**Fig 5:**
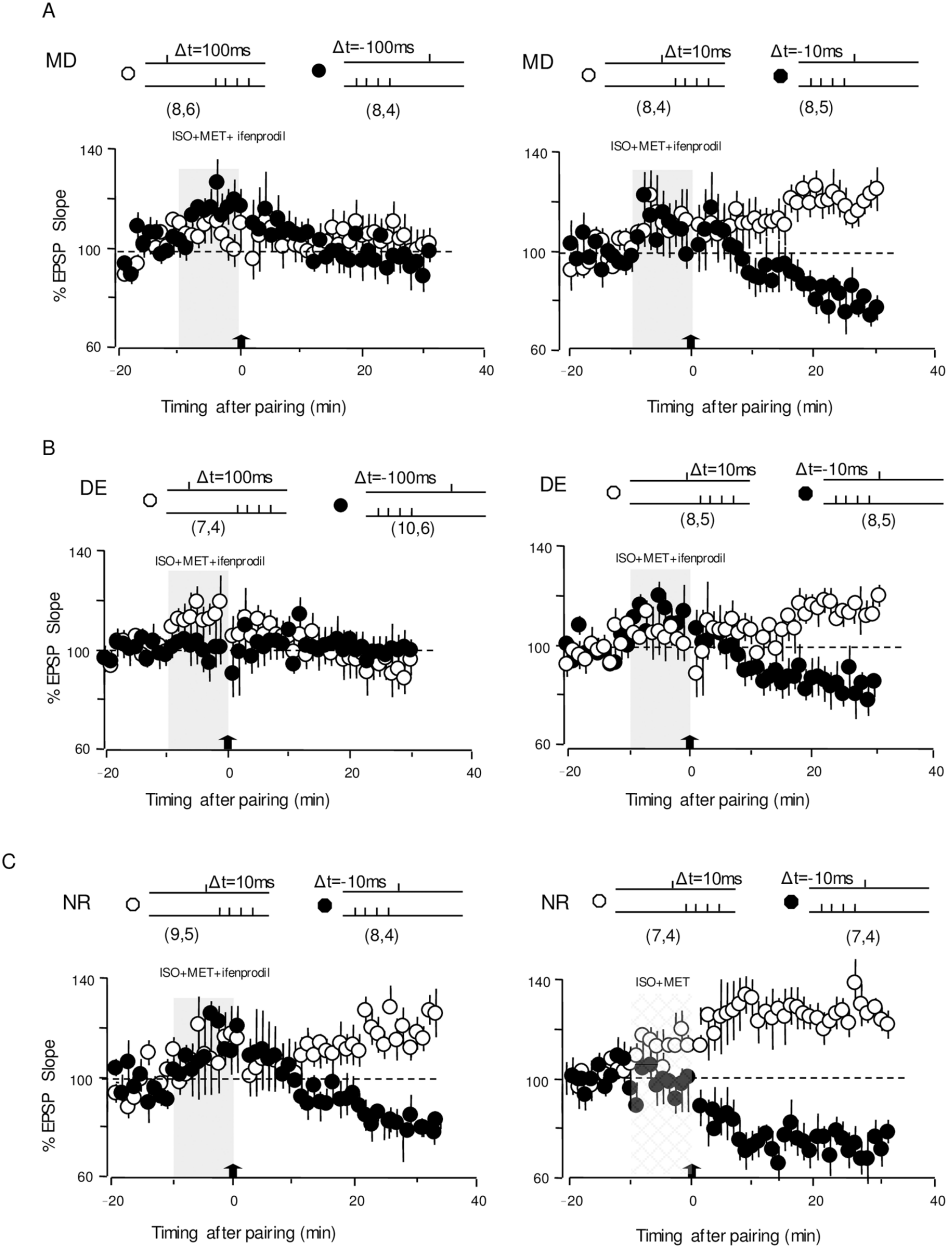
The effect of NR2b-containing NMDAR in the expansion of temporal window for tLTP and tLTD. (A). After brief application of the NR2b specific antagonist Ifenprodil (Ifenprodil: 3mM,mixed up with 10μM isoproterenol and 5 μM Methoxamine,10mins,grey bar), the pairing paradigm (arrow) does not affect EPSPs with long delays (+100ms& -100ms Left). However, Ifenprodil does not block the induction of either tLTP and tLTD with short delays (+10ms&-10ms Right) in cells from MD mice. (B). No tLTP and tLTD are induced by pairing at long delays (+100ms& -100ms Left), but robust tLTP and tLTD elicited at short delays in the presence of ifenprodil in cells from DE mice (3mM ifenprodil, 10μM isoproterenol and 5 μM Methoxamine,10mins, grey bar). (C) In cells from NR mice, ifenprodil cannot block tLTP and tLTD with short delays. (3mM ifenprodil, 10μM isoproterenol and 5 μM Methoxamine,10mins, grey bar). Plotted data is average± SEM. Calibration:10mV,5ms.

In the first round of experiments for MD mice, the pairings (pre-then-post and post-then-pre) were delivered during the end of NR2B antagonist application with Ifenprodil (3 mM) to investigate its effect on tLTP and tLTD induction. Neither of the long-delay (100 ms and -100 ms) stimulus-pairings could elicit tLTP and tLTD in cells from MD with the application of ifenprodil (+100 ms ifenprodil:102.6%±7.8%, n = 8,6, p = 0.68; -100 ms ifenprodil: 95.1%±6.2%, n = 8,4, p = 0.43; Fig 5A). In contrast, robust LTP and LTD, with small changes in amplitude, were induced with short-delay pre-post pairing (10 ms and -10 ms) delivered at the end of NR2B blocker application (+10 ms ifenprodil:121.9%±6.5%, n = 8,4, p = 0.004;-10 ms ifenprodil:81.78%±5.02%, n = 8,5, p = 0.001; Fig 5A). Similar to control NR mice, robust tLTP and tLTD can still be induced after bath application of ifenprodil in NR mice with conditioning by 10 ms and -10 ms pairing stimulation (+10 ms ifenprodil:119.5%±5.6%, n = 9,5, p = 0.002; -10 ms ifenprodil 81.5%±4.8%, n = 8,4, p = 0.0003; Fig 5C; +10 ms control: 123.4±4.2%, n = 7,4; -10 ms control: 77.4±4%, n = 7,4; p<0.001;Fig 5C). Thus, these experiments revealed that the broad temporal window for t-LTP and tLTD in MD mice was shortened by specific NR2B blockade.

**Fig 6:**
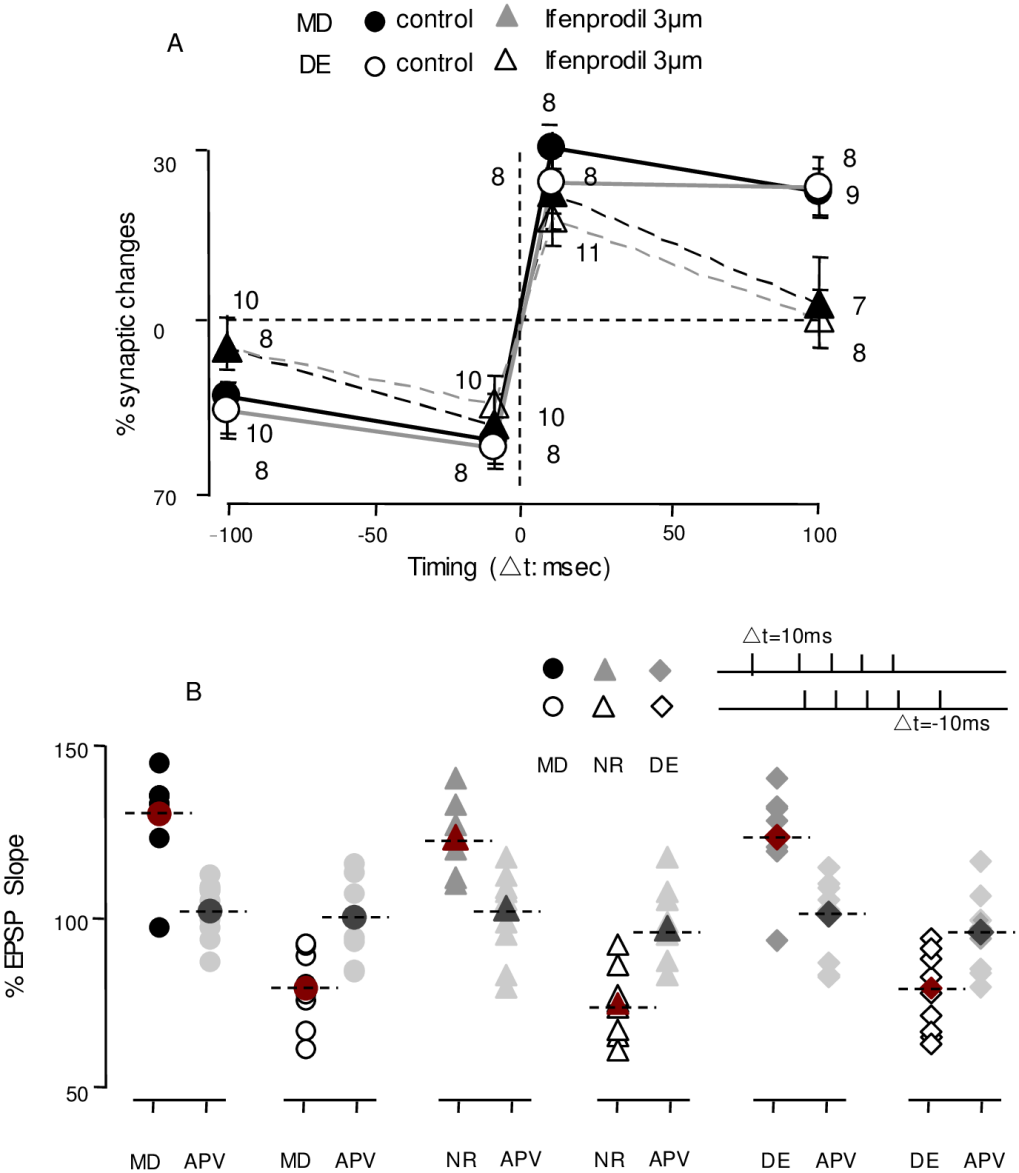
Effect of NR2B-containing NMDARs in the extended temporal window. (A). Changes in the EPSPs induced by pairing sequences with varied intervals in the presence (triangles) or absence of Ifenprodil (circle) in cells from MD(fill circle and triangles) and DE (open circle and triangles). (B)Changes in the EPSPs elicited by pairing with short delays(+10m&-10ms) in the presence of APV in cells from MD,NR,DE.

Subsequently, with a remarkable resemblance of timing window changes for 2-day dark exposure, tLTP and tLTD were elicited reliably with pairing delays of +100 ms and -100 ms at the L4-2/3 synapse after 6-day dark exposure. The prolonged timing window was shortened by ifenprodil application and became closer to the range of the NR STDP temporal window with fewer changes the in short delay as previously described[24] (+100 ms ifenprodil:100.0%±7.1%, n = 7,4, p = 0.94; -100 ms ifenprodil:94.4%±4.7%, n = 10,6, p = 0.2; Fig 5B; +10 ms ifenprodil:118.7%±5.2%, n = 8,5, p = 0.002; -10 ms ifenprodil:85.2%±4.9%, n = 8,5, p = 0.004 Fig 5B). To better clarify the effect of ifenprodil application for tLTP and tLTD elicitation in monocular deprived cells and dark exposed cells, we summarized these results in Fig 6A. The data shown that the antagonist blocked tLTP and tLTD indction with long delays(2-way ANOVA: F[1,27] = 23.51, p<0.001 for DE; F[1,27] = 17.42, p<0.001 for MD), but had little effect on STDP induction with short delays both in cells from MD mice and DE mice(2-way ANOVA: F[1,27] = 0.35, p = 0.55 for DE; 2-way ANOVA: F[1,27] = 0.14, p = 0.7 for MD).

Next, LTP and LTD were attempted using a pairing protocol with 10 ms and -10 ms delays in the presence of APV (50 mM). APV completely blocked the LTP (MD_APV_: 101.7%±3.55%, n = 8,4, p = 0.67; NR_APV_ 102.9%±4.0%, n = 9,5, p = 0.51; DE _APV_ 100.8%±4.4%, n = 9,4, p = 0.38 Fig 6B) and tLTD (MD_APV_: 99.6%±5.1%, n = 8,4, p = 0.93; NR_APV_ 96.5%±4.1%, n = 9,5, p = 0.69; DE_APV_ 95.1%±4.4%, n = 8,5, p = 0.7 Fig 6B) in cells from NR, DE, and MD mice. Taken together, these results support the idea that postsynaptic NMDA receptors are required to participate for STDP induction by the pairing paradigm at synapses from MD mice cortices. The prolonged temporal window was attributed to the high portion of NR2B-containing receptors. This suggests that visual deprivation, including MD and DE, promotes a prolonged STDP timing window due to a high proportion of NR2B-containing postsynaptic NMDA receptors.

## Discussion

As common models for studying experience-dependent plasticity, monocular deprivation and dark exposure have largely been used because of their effects on cortical neurons. In contrast to dark exposure, monocular deprivation degrades image formation but does not impede diffuse light penetration.[35,50–52]. Therefore, there is visually driven activity arising from diffuse light across the closed lid of contralateral eye. Here, we investigated whether the effects of deprivation on timing-dependent LTP and LTD are different based on visual experience. Our data revealed that both monocular deprivation and dark exposure (6 days) extended the temporal integration window for STDP[24]. It is worth noting that the increased fraction of NR2B-containing NMDARs played a principal role in these changes, which was concurrent with a prolonged NMDAR-mediated response duration. Further, our examinations of 6-day sensory deprivation impacts on the NR2A/NR2B ratio revealed a rapid decrease in NR2A rather than an increase in NR2B protein expression. Overall, our data demonstrated that these two types of visual deprivation paradigms shared similar mechanisms for STDP temporal window extension.

As a classic pattern, monocular deprivation during a critical period induces a rapid weakening of responses evoked through the deprived eye followed by a delayed strengthening of responses through the unaffected, intact eye. Indeed, a significant change in the NR2A/B ratio was observed in the deprived visual cortex following 5 and 7 days of deprivation[39]. For this reason, we chose to investigate monocular deprivation over the course of 6 days, which may affect the NMDAR subunit composition in the monocular region of contralateral visual cortex. Although V1M and V1B are closely related cortical structures, the changes in NMDAR subunit composition in V1B, which may alter the properties of visual cortical plasticity, remains to be determined.

In the somatosensory cortex, reduction of the tLTP window was observed in univibrissa-reared mice, where the duration of tLTP was only one-third shorter than the window for tLTD[14]. In contrast to the narrow tLTP window in S1, monocular deprivation prolonged the temporal integration window for timing-dependent LTP. Notably, a prolonged NMDAR-mediated response duration, which was due to the increased fraction of NR2B-containing NMDARs, played a principal role in these changes[24,53,54]. Several lines of evidence support the idea that these mechanisms underlie the facilitating effect of different visual deprivation modalities on tLTP induction. On the one hand, the composition of NMDA receptors changed in electrophysiological measures in the V1M with an increased proportion of NR2b-containing NMDARs. These changes were accompanied by a prolonged excitatory postsynaptic NMDA current decay time course and higher sensitivity to the NR2B subunit-selective antagonist, ifenprodil[55,56]. All of these findings are consistent with the decresed NR2A/NR2B ratio after dark exposure and monocular deprivation supported by previous studies[39,43]. On the other hand, the specific NR2b-NMDAR blocker reshaped the timing window of tLTP elicited by MD or DE into the normal tLTP profile. Furthermore, the rest of LTP was abolished by D-APV application. Third, the available data strongly suggest that visual deprivation, either DE or MD, altered the levels of NMDAR proteins in the synaptoneurosomes, with a decreased NR2A/B protein ratio occurring as a result of decreased NR2A subunit expression of functional synaptic NMDARs. How early experience affects the expression of excitatory receptors is still controversial because they are quite different, depending not only on varied visual fields in the cortex and the duration of visual deprivation[57] but also on the species of animal. However, consensuses have been made that either monocular deprivation or dark exposure generation of lowered cortical activity have similar effects on NR2A and NR2B subunit expression, which is consistent with the findings presented here. It is worth recalling that the temporal window may be reshaped[58] by other mechanisms under monocular deprivation. In addition to the temporal changes in NMDAR glutamate binding[59], the magnitude and localization [60] of dendritic sodium[16] and calcium channels[61,62] are thought to affect the timing requirements and magnitudes of tLTP[63]. The tLTP temporal window could also be expected to vary with the location of the stimulated inputs[16]. More recently, several studies[64] have indicated that NR3A forms complexes with NR1/NR2 decreased unitary channel conductance and increased mean open time compared to NR1/ NR2 complexes, which may influence the t-LTP induction.

The results presented here did not contradict the tLTP profile described in the somatosensory cortex. One property of tLTP elicited from V1 is that it required a neuromodulator as a “prime factor”to activate a neuromodulary receptor for “helping” tLTP induction in the primary visual cortex through kinases activity, phosphates or facilitation of NMDAR currents directly. On the contrary, tLTP was dependent on endogenous levels of neuromodulators in S1. Studies showing that timing-dependent potentiation (tLTP) was induced in developing visual cortices (<P20) younger than those of our experimental subjects[65,66] indicated that developmental mechanisms tightly regulate STDP in the visual cortex. Therefore, the endogenous levels of neuromodulators must be explored at a certain stage of development. Another reasonable explanation is that there was an adaptation in the cortex that allowed response potentiation after 6 days of contralateral eye MD. According to the BCM theory, the reduction in overall cortical activity caused by closing the contralateral eyelid extends the timing window of the modification threshold, thereby facilitating the potentiation of correlated inputs. This theory is also used to interpret the effects of reverse patching on an amblyopic eye.

The broadened tLTD window in MD mice is complicated to explain, but it is quite similar to previous descriptions of tLTD after principal whisker deprivation. There are some differences between these situations. The depression of sensory responses in the primary sensory cortex requires presynaptic and postsynaptic events. Spontaneous activities that are either correlated or uncorrelated with postsynaptic stimulus-driven spiking are required. First, presynaptic NMDAR expression may be required to help induce this form of tLTD. As discussed, a developmental switch contributes to presynaptic and postsynaptic NMDA receptor activity in tLTD. To exclude the possibility that tLTD requires presynaptic NMDARs, the paired-pulse ratio (PPR) was investigated. An unchanged PPR after tLTD and tLTP indicates a postsynaptic NMDAR-dependent mechanism of expression of STDP[19,67]. Second, the postsynaptic firing in deprived S1 was activated by neighboring whiskers, which cannot realistically be simulated in the monocular region of primary visual cortex. In spite of multiple models attempting to interpret how action potentials precede synaptic activation to elicit a LTD-Ca2^+^ signal, postsynaptic NMDAR activation is required for tLTD induction. According to the depolarization model, an increased NMDAR response duration may modestly prolong the tLTD window. Our results suggest that the response duration of postsynaptic NMDARs is one of the precipitating factors for this effect. However, all of the factors that influence the time course of the Ca^2+^ signal may change the temporal window for LTD, including the time course of dendritic repolarization following the back-propagating AP, the types of Ca^2+^ sources in local dendrites, changes in somatic and dendritic excitability, and the process related to the anchoring of NR2B-NMDAR.

Our results demonstrated that synapses of the deprived visual cortex, from either dark exposure or monocular deprivation, possess a higher fraction of NR2B-containing NMDARs. However, the spatial scales of NMDAR compositional changes and the precise roles of specific receptor subunits are controversial. Previous studies have reported that synaptic incorporation of NR2A-containing receptors requires synaptic activity or sensory experience[43,68,69]. However, NR2B-containing receptors are considered to be incorporated in a constitutive manner[70,71]. Our test of the effects of 6-day visual deprivation on subunit expression of NMDA receptors did not reveal any increases in NR2B protein expression with a reduction in NR2A, which shows the dynamic changes between NR2A/NR2B expressions at synapses. These results are consistent with the idea that dark exposure in juvenile animal models causes a rapid increase in NR2B after 2 days of deprivation[24], followed by a decrease in NR2A[39] at later time points. These substantially different modalities of sensory deprivation share the common feature of reducing activities in the visual cortex.

It is generally accepted that critical periods (CPs) occur during mammalian postnatal development when the visual cortex is especially sensitive to visual experience changes. In rodents, the duration of CPs has been widely studied. The CPs begin around the end of the third postnatal week and peak between the fourth and fifth weeks, before beginning to decline at the end of the fifth week. It should be to noted that the peak of CPs for mice is from P29 to P32. However, in this study, the age range for deprivation was slightly young, which may have resulted in a smaller deprivation effect on visual cortical neurons. The difference in NMDAR function observed here was worthy of attention because it may be able to self-regulate at a more flexible stage.

In general, we proposed that the tLTP and tLTD timing windows of MD and DE mice were broader than normal controls in the primary visual cortex. In our studies, these substantially different modes of sensory deprivation shared the common effect of reducing activity in the visual cortex by increasing the synaptic fraction of NR2b-containing NMDAR, which is known to expand the temporal window for STDP with a spike-timing plasticity protocol together with neuromodulator stimulation. Extended timing windows may be intriguing potential mechanisms for explaining cortical remapping in juvenile and adult mammals after sensory experience manipulations, such as reverse patching, environmental enrichment [72] or dark exposure[73], accompanied by a high fraction of NR2B-containing NMDAR.

## Supporting information

S1 Fig Both MD and DE extend the integration time for the tLTD and tLTP induction for 60 mins when delays are 100ms and -100ms. Fig A in S1 fig:In cells from DE mice, isoproterenol and methoxamine promote the induction of tLTP and tLTD also maintain for 60 minutes when delay are 100 ms and -100ms. Fig B in S1 fig:In cells from MD mice, tLTP and tLTD can be maintained with isoproterenol and methoxamine when delays are 100ms and -100ms. Plotted data is average ± SEM; Calibration: 6 mV, 10 ms.
(TIF)

## References

[1] Hubel DH, Wiesel TN. The period of susceptibility to the physiological effects of unilateral eye closure in kittens. J Physiol. 1970;206(2):419–36. ; 5498493 10.1113/jphysiol.1970.sp009022 PMC1348655

[2] Fagiolini M, Pizzorusso T, Berardi N, Domenici L, Maffei L. Functional postnatal development of the rat primary visual cortex and the role of visual experience: dark rearing and monocular deprivation. Vision research. 1994;34(6):709–20. . 8160387 10.1016/0042-6989(94)90210-0

[3] Cooke SF, Bear MF. Visual experience induces long-term potentiation in the primary visual cortex. J Neurosci. 30(48):16304–13. Epub 2010/12/03. doi: 10.1523/JNEUROSCI.4333-10.2010 ; 21123576 PMC3078625

[4] Rittenhouse CD, Shouval HZ, Paradiso MA, Bear MF. Monocular deprivation induces homosynaptic long-term depression in visual cortex. Nature. 1999;397(6717):347–50. doi: 10.1038/16922 . 9950426

[5] Bienenstock EL, Cooper LN, Munro PW. Theory for the development of neuron selectivity: orientation specificity and binocular interaction in visual cortex. J Neurosci. 1982;2(1):32–48. Epub 1982/01/01. . 7054394 10.1523/JNEUROSCI.02-01-00032.1982 PMC6564292

[6] Heynen AJ, Yoon BJ, Liu CH, Chung HJ, Huganir RL, Bear MF. Molecular mechanism for loss of visual cortical responsiveness following brief monocular deprivation. Nat Neurosci. 2003;6(8):854–62. Epub 2003/07/30. doi: 10.1038/nn1100 . 12886226

[7] Katz LC, Shatz CJ. Synaptic activity and the construction of cortical circuits. Science. 1996;274(5290):1133–8. . 8895456 10.1126/science.274.5290.1133

[8] Kirkwood A, Bear MF. Homosynaptic long-term depression in the visual cortex. J Neurosci. 1994;14(5 Pt 2):3404–12. . 8182481 10.1523/JNEUROSCI.14-05-03404.1994 PMC6577491

[9] Froemke RC, Dan Y. Spike-timing-dependent synaptic modification induced by natural spike trains. Nature. 2002;416(6879):433–8. doi: 10.1038/416433a . 11919633

[10] Luz Y, Shamir M. The effect of STDP temporal kernel structure on the learning dynamics of single excitatory and inhibitory synapses. PLoS One. 2014;9(7):e101109. doi: 10.1371/journal.pone.0101109 . 24999634 PMC4085044

[11] Caporale N, Dan Y. Spike timing-dependent plasticity: a Hebbian learning rule. Annu Rev Neurosci. 2008;31:25–46. doi: 10.1146/annurev.neuro.31.060407.125639 . 18275283

[12] Richards BA, Aizenman CD, Akerman CJ. In vivo spike-timing-dependent plasticity in the optic tectum of Xenopus laevis. Front Synaptic Neurosci. 2:7. Epub 2010/01/01. doi: 10.3389/fnsyn.2010.00007 . 21423493 PMC3059697

[13] Allen CB, Celikel T, Feldman DE. Long-term depression induced by sensory deprivation during cortical map plasticity in vivo. Nat Neurosci. 2003;6(3):291–9. doi: 10.1038/nn1012 . 12577061

[14] Feldman DE. Timing-based LTP and LTD at vertical inputs to layer II/III pyramidal cells in rat barrel cortex. Neuron. 2000;27(1):45–56. . 10939330 10.1016/s0896-6273(00)00008-8

[15] Celikel T, Szostak VA, Feldman DE. Modulation of spike timing by sensory deprivation during induction of cortical map plasticity. Nat Neurosci. 2004;7(5):534–41. doi: 10.1038/nn1222 . 15064767 PMC3082358

[16] Froemke RC, Tsay IA, Raad M, Long JD, Dan Y. Contribution of individual spikes in burst-induced long-term synaptic modification. J Neurophysiol. 2006;95(3):1620–9. doi: 10.1152/jn.00910.2005 . 16319206

[17] Zilberter M, Holmgren C, Shemer I, Silberberg G, Grillner S, Harkany T, et al Input specificity and dependence of spike timing-dependent plasticity on preceding postsynaptic activity at unitary connections between neocortical layer 2/3 pyramidal cells. Cereb Cortex. 2009;19(10):2308–20. doi: 10.1093/cercor/bhn247 ; 19193711 PMC2742592

[18] Corlew R, Wang Y, Ghermazien H, Erisir A, Philpot BD. Developmental switch in the contribution of presynaptic and postsynaptic NMDA receptors to long-term depression. J Neurosci. 2007;27(37):9835–45. doi: 10.1523/JNEUROSCI.5494-06.2007 ; 17855598 PMC2905826

[19] Seol GH, Ziburkus J, Huang S, Song L, Kim IT, Takamiya K, et al Neuromodulators control the polarity of spike-timing-dependent synaptic plasticity. Neuron. 2007;55(6):919–29. Epub 2007/09/21. doi: 10.1016/j.neuron.2007.08.013 ; 17880895 PMC2756178

[20] Larsen RS, Rao D, Manis PB, Philpot BD. STDP in the Developing Sensory Neocortex. Front Synaptic Neurosci. 2010;2:9. doi: 10.3389/fnsyn.2010.00009 ; 21423495 PMC3059680

[21] Gabbott PL, Stewart MG. Visual deprivation alters dendritic bundle architecture in layer 4 of rat visual cortex. Neuroscience. 207:65–77. Epub 2012/01/25. doi: 10.1016/j.neuroscience.2012.01.003 . 22269141

[22] Tropea D, Majewska AK, Garcia R, Sur M. Structural dynamics of synapses in vivo correlate with functional changes during experience-dependent plasticity in visual cortex. J Neurosci. 30(33):11086–95. Epub 2010/08/20. doi: 10.1523/JNEUROSCI.1661-10.2010 ; 20720116 PMC2932955

[23] Carlson M, Hubel DH, Wiesel TN. Effects of monocular exposure to oriented lines on monkey striate cortex. Brain Res. 1986;390(1):71–81. Epub 1986/02/01. . 3948033 10.1016/0165-3806(86)90153-7

[24] Guo Y, Huang S, de Pasquale R, McGehrin K, Lee HK, Zhao K, et al Dark exposure extends the integration window for spike-timing-dependent plasticity. J Neurosci. 32(43):15027–35. Epub 2012/10/27. doi: 10.1523/JNEUROSCI.2545-12.2012 ; 23100424 PMC3496177

[25] Huang S, Hokenson K, Bandyopadhyay S, Russek SJ, Kirkwood A. Brief Dark Exposure Reduces Tonic Inhibition in Visual Cortex. J Neurosci. 35(48):15916–20. Epub 2015/12/04. doi: 10.1523/JNEUROSCI.1813-15.2015 ; 26631472 PMC4666916

[26] Zhou J, Baker DH, Simard M, Saint-Amour D, Hess RF. Short-term monocular patching boosts the patched eye's response in visual cortex. Restor Neurol Neurosci. 33(3):381–7. Epub 2015/09/28. doi: 10.3233/RNN-140472 . 26410580 PMC4923712

[27] Lunghi C, Berchicci M, Morrone MC, Di Russo F. Short-term monocular deprivation alters early components of visual evoked potentials. J Physiol. 593(19):4361–72. Epub 2015/06/30. doi: 10.1113/JP270950 ; 26119530 PMC4594246

[28] Mainardi M, Landi S, Berardi N, Maffei L, Pizzorusso T. Reduced responsiveness to long-term monocular deprivation of parvalbumin neurons assessed by c-Fos staining in rat visual cortex. PLoS One. 2009;4(2):e4342. doi: 10.1371/journal.pone.0004342 ; 19194492 PMC2632740

[29] Kang E, Durand S, LeBlanc JJ, Hensch TK, Chen C, Fagiolini M. Visual acuity development and plasticity in the absence of sensory experience. J Neurosci. 33(45):17789–96. Epub 2013/11/08. doi: 10.1523/JNEUROSCI.1500-13.2013 ; 24198369 PMC3818552

[30] Nahmani M, Turrigiano GG. Deprivation-induced strengthening of presynaptic and postsynaptic inhibitory transmission in layer 4 of visual cortex during the critical period. J Neurosci. 34(7):2571–82. Epub 2014/02/14. doi: 10.1523/JNEUROSCI.4600-13.2014 ; 24523547 PMC3921427

[31] Berardi N, Pizzorusso T, Ratto GM, Maffei L. Molecular basis of plasticity in the visual cortex. Trends Neurosci. 2003;26(7):369–78. Epub 2003/07/10. doi: 10.1016/S0166-2236(03)00168-1 . 12850433

[32] Yoneda T, Kameyama K, Esumi K, Daimyo Y, Watanabe M, Hata Y. Developmental and visual input-dependent regulation of the CB1 cannabinoid receptor in the mouse visual cortex. PLoS One. 2013;8(1):e53082. doi: 10.1371/journal.pone.0053082 ; 23308141 PMC3540079

[33] Leventhal AG, Hirsch HV. Effects of early experience upon orientation sensitivity and binocularity of neurons in visual cortex of cats. Proceedings of the National Academy of Sciences. 1977;74(3):1272–6. 10.1073/pnas.74.3.1272 PMC430666 265570

[34] Leventhal AG, Hirsch HV. Receptive-field properties of different classes of neurons in visual cortex of normal and dark-reared cats. J Neurophysiol. 1980;43(4):1111–32. . 7359177 10.1152/jn.1980.43.4.1111

[35] Blais BS, Frenkel MY, Kuindersma SR, Muhammad R, Shouval HZ, Cooper LN, et al Recovery from monocular deprivation using binocular deprivation. J Neurophysiol. 2008;100(4):2217–24. doi: 10.1152/jn.90411.2008 ; 18650311 PMC2576197

[36] Whitt JL, Petrus E, Lee HK. Experience-dependent homeostatic synaptic plasticity in neocortex. Neuropharmacology. 2014;78(3):45–54. 23466332 10.1016/j.neuropharm.2013.02.016 PMC3796008

[37] Wiesel TN. Postnatal development of the visual cortex and the influence of environment. Nature. 1982;299(5884):583–91. . 6811951 10.1038/299583a0

[38] Gordon JA, Stryker MP. Experience-dependent plasticity of binocular responses in the primary visual cortex of the mouse. J Neurosci. 1996;16(10):3274–86. . 8627365 10.1523/JNEUROSCI.16-10-03274.1996 PMC6579137

[39] Chen WS, Bear MF. Activity-dependent regulation of NR2B translation contributes to metaplasticity in mouse visual cortex. Neuropharmacology. 2007;52(1):200–14. Epub 2006/08/10. doi: 10.1016/j.neuropharm.2006.07.003 . 16895734

[40] Huang S, Trevino M, He K, Ardiles A, Pasquale R, Guo Y, et al Pull-push neuromodulation of LTP and LTD enables bidirectional experience-induced synaptic scaling in visual cortex. Neuron. 2012;73(3):497–510. doi: 10.1016/j.neuron.2011.11.023 ; 22325202 PMC3373163

[41] Nataraj K, Turrigiano G. Regional and temporal specificity of intrinsic plasticity mechanisms in rodent primary visual cortex. Journal of Neuroscience the Official Journal of the Society for Neuroscience. 2011;31(49):17932–40. 10.1523/JNEUROSCI.4455-11.2011 PMC3272675 22159108

[42] Hollingsworth EB, McNeal ET, Burton JL, Williams RJ, Daly JW, Creveling CR. Biochemical characterization of a filtered synaptoneurosome preparation from guinea pig cerebral cortex: cyclic adenosine 3':5'-monophosphate-generating systems, receptors, and enzymes. J Neurosci. 1985;5(8):2240–53. . 2991484 10.1523/JNEUROSCI.05-08-02240.1985 PMC6565304

[43] Quinlan EM, Olstein DH, Bear MF. Bidirectional, experience-dependent regulation of N-methyl-D-aspartate receptor subunit composition in the rat visual cortex during postnatal development. Proc Natl Acad Sci U S A. 1999;96(22):12876–80. Epub 1999/10/27. ; 10536016 10.1073/pnas.96.22.12876 PMC23143

[44] Frenkel MY, Bear MF. How monocular deprivation shifts ocular dominance in visual cortex of young mice. Neuron. 2004;44(6):917–23. doi: 10.1016/j.neuron.2004.12.003 . 15603735

[45] Larsen RS, Smith IT, Miriyala J, Han JE, Corlew RJ, Smith SL, et al Synapse-specific control of experience-dependent plasticity by presynaptic NMDA receptors. Neuron. 2014;83(4):879–93. doi: 10.1016/j.neuron.2014.07.039 ; 25144876 PMC4181612

[46] Rodriguez-Moreno A, Paulsen O. Spike timing-dependent long-term depression requires presynaptic NMDA receptors. Nat Neurosci. 2008;11(7):744–5. doi: 10.1038/nn.2125 . 18516036

[47] Philpot BD, Sekhar AK, Shouval HZ, Bear MF. Visual experience and deprivation bidirectionally modify the composition and function of NMDA receptors in visual cortex. Neuron. 2001;29(1):157–69. . 11182088 10.1016/s0896-6273(01)00187-8

[48] Carmignoto G, Vicini S. Activity-dependent decrease in NMDA receptor responses during development of the visual cortex. Science. 1992;258(5084):1007–11. . 1279803 10.1126/science.1279803

[49] Koester HJ, Johnston D. Target cell-dependent normalization of transmitter release at neocortical synapses. Science. 2005;308(5723):863–6. doi: 10.1126/science.1100815 . 15774725

[50] Maffei A, Nataraj K, Nelson SB, Turrigiano GG. Potentiation of cortical inhibition by visual deprivation. Nature. 2006;443(7107):81–4. doi: 10.1038/nature05079 . 16929304

[51] Maffei A, Turrigiano GG. Multiple modes of network homeostasis in visual cortical layer 2/3. J Neurosci. 2008;28(17):4377–84. doi: 10.1523/JNEUROSCI.5298-07.2008 ; 18434516 PMC2655203

[52] He K, Petrus E, Gammon N, Lee HK. Distinct sensory requirements for unimodal and cross-modal homeostatic synaptic plasticity. J Neurosci. 2012;32(25):8469–74. doi: 10.1523/JNEUROSCI.1424-12.2012 ; 22723686 PMC3444293

[53] Kirkwood A, Rioult MC, Bear MF. Experience-dependent modification of synaptic plasticity in visual cortex. Nature. 1996;381(6582):526–8. Epub 1996/06/06. doi: 10.1038/381526a0 . 8632826

[54] Philpot BD, Espinosa JS, Bear MF. Evidence for altered NMDA receptor function as a basis for metaplasticity in visual cortex. J Neurosci. 2003;23(13):5583–8. . 12843259 10.1523/JNEUROSCI.23-13-05583.2003 PMC6741231

[55] Liu XB, Murray KD, Jones EG. Switching of NMDA receptor 2A and 2B subunits at thalamic and cortical synapses during early postnatal development. J Neurosci. 2004;24(40):8885–95. doi: 10.1523/JNEUROSCI.2476-04.2004 . 15470155 PMC6729956

[56] Mierau SB, Meredith RM, Upton AL, Paulsen O. Dissociation of experience-dependent and -independent changes in excitatory synaptic transmission during development of barrel cortex. Proc Natl Acad Sci U S A. 2004;101(43):15518–23. doi: 10.1073/pnas.0402916101 ; 15492224 PMC524435

[57] Beston BR, Jones DG, Murphy KM. Experience-dependent changes in excitatory and inhibitory receptor subunit expression in visual cortex. Front Synaptic Neurosci. 2010;2:138. doi: 10.3389/fnsyn.2010.00138 ; 21423524 PMC3059668

[58] Shouval HZ, Bear MF, Cooper LN. A unified model of NMDA receptor-dependent bidirectional synaptic plasticity. Proc Natl Acad Sci U S A. 2002;99(16):10831–6. doi: 10.1073/pnas.152343099 ; 12136127 PMC125058

[59] Laurie DJ, Seeburg PH. Ligand affinities at recombinant N-methyl-D-aspartate receptors depend on subunit composition. European journal of pharmacology. 1994;268(3):335–45. 7528680 10.1016/0922-4106(94)90058-2

[60] Sjostrom PJ, Hausser M. A cooperative switch determines the sign of synaptic plasticity in distal dendrites of neocortical pyramidal neurons. Neuron. 2006;51(2):227–38. doi: 10.1016/j.neuron.2006.06.017 . 16846857 PMC7616902

[61] Nevian T, Sakmann B. Spine Ca2+ signaling in spike-timing-dependent plasticity. J Neurosci. 2006;26(43):11001–13. doi: 10.1523/JNEUROSCI.1749-06.2006 . 17065442 PMC6674669

[62] Kampa BM, Letzkus JJ, Stuart GJ. Requirement of dendritic calcium spikes for induction of spike-timing-dependent synaptic plasticity. J Physiol. 2006;574(Pt 1):283–90. doi: 10.1113/jphysiol.2006.111062 ; 16675489 PMC1817800

[63] Hoffman DA, Magee JC, Colbert CM, Johnston D. K+ channel regulation of signal propagation in dendrites of hippocampal pyramidal neurons. Nature. 1997;387(6636):869 10.1038/43119 9202119

[64] Sasaki YF, Rothe T, Premkumar LS, Das S, Cui J, Talantova MV, et al Characterization and comparison of the NR3A subunit of the NMDA receptor in recombinant systems and primary cortical neurons. J Neurophysiol. 2002;87(4):2052–63. doi: 10.1152/jn.00531.2001 . 11929923

[65] Sjostrom PJ, Turrigiano GG, Nelson SB. Rate, timing, and cooperativity jointly determine cortical synaptic plasticity. Neuron. 2001;32(6):1149–64. . 11754844 10.1016/s0896-6273(01)00542-6

[66] Markram H, Lubke J, Frotscher M, Sakmann B. Regulation of synaptic efficacy by coincidence of postsynaptic APs and EPSPs. Science. 1997;275(5297):213–5. . 8985014 10.1126/science.275.5297.213

[67] Huang S, Huganir RL, Kirkwood A. Adrenergic gating of Hebbian spike-timing-dependent plasticity in cortical interneurons. J Neurosci. 2013;33(32):13171–8. doi: 10.1523/JNEUROSCI.5741-12.2013 ; 23926270 PMC3735889

[68] Philpot BD, Cho KK, Bear MF. Obligatory role of NR2A for metaplasticity in visual cortex. Neuron. 2007;53(4):495–502. doi: 10.1016/j.neuron.2007.01.027 ; 17296552 PMC1847797

[69] Quinlan EM, Philpot BD, Huganir RL, Bear MF. Rapid, experience-dependent expression of synaptic NMDA receptors in visual cortex in vivo. Nat Neurosci. 1999;2(4):352–7. Epub 1999/04/16. doi: 10.1038/7263 . 10204542

[70] Storey GP, Opitz-Araya X, Barria A. Molecular determinants controlling NMDA receptor synaptic incorporation. J Neurosci. 2011;31(17):6311–6. doi: 10.1523/JNEUROSCI.5553-10.2011 ; 21525271 PMC3092540

[71] Barria A, Malinow R. Subunit-specific NMDA receptor trafficking to synapses. Neuron. 2002;35(2):345–53. . 12160751 10.1016/s0896-6273(02)00776-6

[72] Sale A, Maya Vetencourt JF, Medini P, Cenni MC, Baroncelli L, De Pasquale R, et al Environmental enrichment in adulthood promotes amblyopia recovery through a reduction of intracortical inhibition. Nat Neurosci. 2007;10(6):679–81. doi: 10.1038/nn1899 . 17468749

[73] He HY, Hodos W, Quinlan EM. Visual deprivation reactivates rapid ocular dominance plasticity in adult visual cortex. J Neurosci. 2006;26(11):2951–5. doi: 10.1523/JNEUROSCI.5554-05.2006 . 16540572 PMC6673977

