# Supplementary figures and images for: Timing-dependent LTP and LTD in mouse primary visual cortex following different visual deprivation models
Source: PLoS One. 2017 May 17;12(5):e0176603. doi: 10.1371/journal.pone.0176603 (PMC5435181; doi:10.1371/journal.pone.0176603)

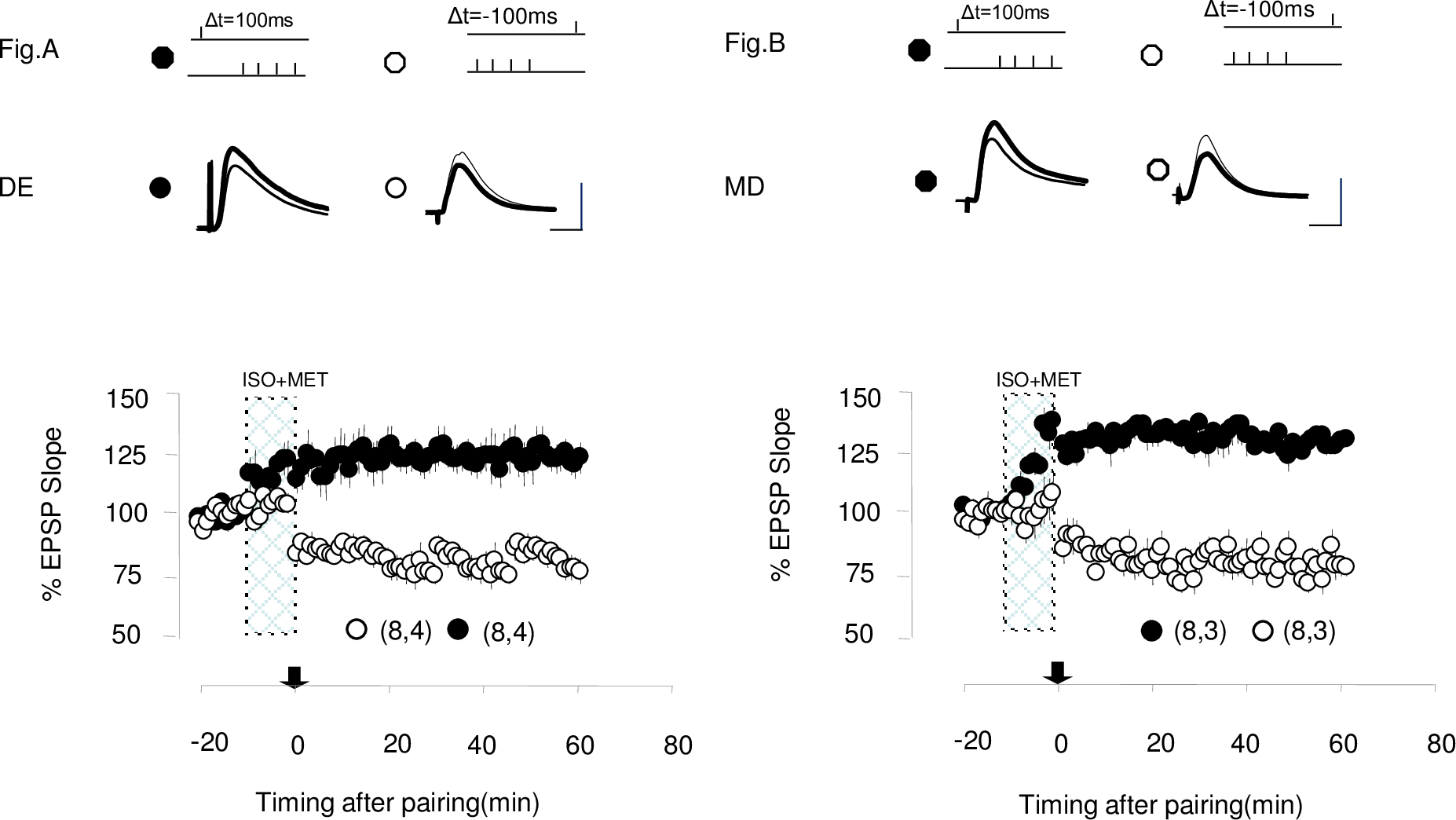

Supplement: S1 Fig — Fig A in S1 fig:In cells from DE mice, isoproterenol and methoxamine promote the induction of tLTP and tLTD also maintain for 60 minutes when delay are 100 ms and -100ms. Fig B in S1 fig:In cells from MD mice, tLTP and tLTD can be maintained with isoproterenol and methoxamine when delays are 100ms and -100ms. Plotted data is average ± SEM; Calibration: 6 mV, 10 ms. (TIF) [file pone.0176603.s001.tif]
